# Functional identification of the DNA packaging terminase from* Pseudomonas aeruginosa* phage PaP3

**DOI:** 10.1007/s00705-012-1409-5

**Published:** 2012-07-22

**Authors:** Xiaodong Shen, Ming Li, Yijun Zeng, Xiaomei Hu, Yinling Tan, Xiancai Rao, Xiaolin Jin, Shu Li, Junmin Zhu, Kebin Zhang, Fuquan Hu

**Affiliations:** 1 Department of Microbiology, Third Military Medical University, Chongqing, China; 2 Department of Biochemistry and Molecular Biology, Third Military Medical University, Chongqing, China; 3 Center of Medical Experiment & Technology of Xinqiao Hospital, Third Military Medical University, Chongqing, China

## Abstract

Terminase proteins are responsible for DNA recognition and initiation of DNA packaging in phages. We previously reported the genomic sequence of a temperate *Pseudomonas aeruginosa* phage, PaP3, and determined its precise integration site in the host bacterial chromosome. In this study, we present a detailed functional identification of the DNA packaging terminase for phage PaP3. The purified large subunit p03 was demonstrated to possess ATPase and nuclease activities, as well as the ability to bind to specific DNA when it is unassembled. In addition, a small terminase subunit (p01) of a new type was found and shown to bind specifically to *cos*-containing DNA and stimulate the *cos*-cleavage and ATPase activities of p03. The results presented here suggest that PaP3 utilizes a typical *cos* site mechanism for DNA packaging and provide a first step towards understanding the molecular mechanism of the PaP3 DNA packaging reaction.

**Electronic supplementary material:**

The online version of this article (doi:10.1007/s00705-012-1409-5) contains supplementary material, which is available to authorized users.

## Introduction

In most double-stranded DNA (dsDNA) bacteriophages and eukaryotic DNA viruses, such as adenovirus and herpesvirus, a key step in virion assembly is the packaging of the viral genome into a preformed empty capsid by the action of an ATP-powered molecular motor [1, 6, 24, 25, 27]. This packaging process is initiated by recognition and endonucleolytic cleavage of viral concatemeric DNA. Concatemeric DNA, which consists of head-to-tail unit-length molecules, is generally produced via recombination [21] or rolling-circle replication [34, 37]. Next, the cleaved DNA end is linked to the portal vertex of the empty prohead through specific interactions between the terminase and the portal protein [19, 20, 38, 41]. Thus, a packaging motor is assembled, which drives directional translocation of DNA into the prohead, powered by the energy of ATP hydrolysis. When the viral head has been filled with one (*cos* phages) or slightly more than one (*pac* phages) genome length, the DNA is cut again, and the packed head attaches the neck and tail components to complete the assembly of an infectious virion [1, 2, 33]. Finally, the undocked terminase reassociates with another empty prohead to continue head filling in a processive manner [9, 27, 33].

Terminase is a key component of this highly dynamic process. The terminase enzyme is normally a heteromultimer composed of one large and one small subunit. The small subunit can specifically bind the viral DNA and is hypothesized to be involved in recognition of the viral genome substrate. The large subunit, which is the main component of the terminase holoenzyme, is required for DNA cleavage to generate single genome-length molecules, linkage of cleaved DNA to the connector, and translocation of DNA into the empty prohead [4, 27]. Because the packaging reaction catalyzed by terminase is highly specific, terminase enzymes represent ideal models to investigate protein-protein and nucleotide-protein interactions.

Our laboratory is interested in the genetic and biochemical basis of phage-bacteria interactions and, in particular, the application of genetic remodeling in the improvement of phage therapy. We previously isolated and identified three new strains of *Pseudomonas aeruginosa* phages from our affiliated hospital sewage and designated them as PaP1, PaP2, and PaP3. PaP1 is virulent, while PaP2 and PaP3 are both temperate phages. Recently, we determined the complete nucleotide sequence of the PaP3 genome (GenBank accession number NC_004466) and discovered the mechanism by which it is integrated into the host bacterial chromosome [35]. In the current study, we identified two important genes encoding PaP3 terminase subunits that are required for the DNA packaging process.

## Materials and methods

### Bacteria, phage, and plasmids

The phage, bacterial strains, and plasmids used in this study are listed in Table 1. *P. aeruginosa* phage PaP3 was propagated on *P. aeruginosa* strain PA1 which belongs to the International Antigenic Typing System (IATS) serotype 6. All bacterial cultures were grown in Luria-Bertani (LB) medium or on 1.5 % agar plates at 37 °C. If required, 100 μg/ml of ampicillin (Amp) (Sigma) was added for cloning procedures.

**Table 1:** Phage, bacterial strains, and plasmids used in this study

Strains/plasmids	Characteristics*	Source/reference
**Strains**		
**Phage strain**		
PaP3	*P. aeruginosa* phage isolated from hospital sewage	Lab collection
**Bacterial strains**		
*P. aeruginosa* PA1	Clinical isolate of *P. aeruginosa*, serotype 6	Lab collection
*E. coli* DH5α	Cloning host for maintaining recombinant plasmids	Lab collection
*E. coli* BL21(DE3)	Expression host for recombinant protein production	Lab collection
**Plasmids**		
pMD™18-T	T-cloning vector; Amp^R^	TaKaRa
pMD-*cos*	Derivative of pMD™18-T with a cloned 239-bp PCR product containing the *cos* end sequence of PaP3	This work
pET-22b(+)	C-terminal His tag fusion expression vector; Amp^R^	Novagen
pQE-31	N-terminal His tag fusion expression vector; Amp^R^	Qiagen
pQE31-*p01*	Derivative of pQE-31 containing the PaP3 terminase small subunit coding gene *orf1*	This work
pET22b-*p03*	Derivative of pET-22b(+) containing the PaP3 terminase large subunit coding gene *orf3*	This work

* Amp^R^, ampicillin resistant

### Expression and purification of terminase proteins

The *orf1* gene was PCR-amplified from PaP3 genomic DNA using primers *p01*-F and *p01*-R and cloned into the *Bam*H I/*Hin*d III sites of the pQE-31 expression vector, creating a fusion protein with an N-terminal His_6_ tag. Plasmid pET22b-*p03* was generated by inserting *orf3* PCR products (amplified with primer pair *p03*-F/*p03*-R) into the *Nde* I/*Xho* I sites of the pET-22b(+) vector, creating a C-terminally His_6_-tagged fusion protein. The sequences of the primers used are listed in Table 2. *E. coli* BL21 (DE3) cells harboring the recombinant expression vectors were grown overnight in 50 ml LB broth containing ampicillin at 37 °C. A 1:100 dilution of the overnight culture was used to inoculate 2 L of fresh LB medium containing ampicillin, which was then shaken at 25 °C to an OD_600_ of 0.6. Isopropyl-β-D-thiogalactopyranoside (IPTG) was added to a final concentration of 1 mM to induce overexpression of the recombinant proteins. Following 5 h of induction, the cells were harvested by centrifugation at 6,000 g for 10 min, and the pellet was resuspended in a lysis buffer containing 20 mM Tris-HCl (pH 8.0), 50 mM NaCl, and 5 % glycerol. Lysis was completed by sonication on ice, and the soluble fractions were collected by centrifugation and loaded onto a Ni-NTA affinity column (QIAGEN) that had been equilibrated with cell lysis buffer. The His_6_-tagged proteins were eluted with a gradient of 20-500 mM imidazole and then purified by Superdex-75 big gel filtration chromatography. Peak elution fractions were analyzed by electrophoresis on 10 % (p03) or 15 % (p01) SDS-PAGE followed by Coomassie blue staining. Fractions containing pure proteins were pooled and concentrated in an Amicon apparatus (Millipore) with a 10-kDa molecular weight cutoff membrane and then stored in 0.1-ml aliquots at −80 °C. The concentrations of proteins were determined with a BCA protein assay kit (Pierce) according to the manufacturer’s protocol.

**Table 2:** Primers used in this study for PCR and gene expression analysis

Primer	Sequence (5′-3′)	Function
*cos*-F	GAGCCTGAGTCATGGTCGTTTCAT	Amplification of the *cos*239 fragment
*cos*-R	GATGGGTTAGTGTCGAAGGCTTAG	
*cos*239-F	ATACTCCCCGTCGCGCTTGAACCA	Amplification of the *cos*239dn fragment
*cos*239-R	AGGGTTGACAAGGCAAGCCCACGG	
*p01*-F	GGATCCAATGTCAGACGAAAAGGT (*Bam*H I)	*orf1* expression
*p01*-R	AAGCTTAGCGGTCGGGAAAGAAA (*Hin*d III)	
*p03*-F	CATATGGATACCCAAGAGCGGTTG (*Nde* I)	*orf3* expression
*p03*-R	CTCGAGGACAATACTCCCAAACCA (*Xho* I)	

### *In vitro cos* cleavage assays

A 239-bp fragment containing the PaP3 *cos* site (designated *cos*239) was amplified from the PaP3 genome using PCR primers *cos*-F and *cos*-R. The resulting PCR products were cloned into pMD18-T, creating pMD-*cos*. The plasmid pMD-*cos* (10 nM) was used as substrate DNA and was incubated with the proteins of interest in a reaction buffer containing 50 mM Tris-HCl (pH 8.0), 10 mM MgCl_2_, and 50 mM NaCl at 37 °C for 60 min. Acetylated BSA was used as a negative protein control. The *cos* cleavage reactions were terminated by the addition of EDTA to a final concentration of 20 mM, and the samples were electrophoresed on 0.9 % (w/v) agarose gels followed by ethidium bromide staining. Gel images were captured digitally, and the amount of *cos* cleavage was determined by analyzing band intensities quantified with Quantity One software (Bio-Rad). The yield of cleaved, linearized (L) DNA was calculated after correction for the relative fluorescence of the L form of DNA to the covalently closed circular (CCC) plasmid DNA.

### DNA binding assays

To evaluate the DNA binding ability of the small terminase subunit via electrophoretic mobility shift assays (EMSAs), the abovementioned *cos*239 fragment was used as substrate DNA, and the immediately downstream 239-bp segment (designated *cos*239dn) was used as a nonspecific DNA control. The DNA substrate used to analyze the binding activity of the large subunit of PaP3 terminase was the 20-bp *cos* end sequence of PaP3. A 24-bp non-*cos* DNA (5′-GCACTGCAGTAACTTGTCAGTCAT-3′) was served as a nonspecific control. Purified proteins of interest were mixed with various 5′-end biotin-labeled DNAs (1.5 μM) in 20 μl buffer containing 50 mM Tris-HCl (pH 8.0), 50 mM NaCl, 10 mM MgCl_2_, and 5 % glycerol. The DNA/protein binding reactions were incubated at 37 °C for 30 min and terminated by adding EDTA to a final concentration of 20 mM. All mixtures were loaded and separated on 5 % non-denaturing PAGE gels in 0.5 × TBE buffer and then transferred onto Hybond-N + nylon membranes (Amersham Pharmacia). The biotin end-labeled DNAs were detected using a streptavidin–horseradish peroxidase conjugate and a chemiluminescent substrate developed for the LightShift Chemiluminescent EMSA Kit (Pierce) according to the standard protocol. The signals were then detected with X-ray films.

### ATPase assays

The purified p03 or p01 or the control buffer was incubated in a 20-μl reaction mixture containing 5 μCi of [γ-^32^P]ATP (specific activity 3000 Ci/mmol, GE Healthcare) at 37 °C in ATPase buffer (50 mM Tris-HCl, pH 7.5, 0.1 M NaCl, and 5 mM MgCl_2_) for 30 min. The ATP hydrolysis reactions were terminated by the addition of EDTA to a final concentration of 50 mM, and the products were separated by thin-layer chromatography on PEI plates (Sigma). Phosphorimaging (Storm 820, Molecular Dynamics) was used for data quantification.

## Results

In a previous report, we determined the complete genomic sequence of phage PaP3 [35]. PaP3 contains a linear 45,503-bp dsDNA chromosome with 5′ protruding cohesive (*cos*) ends (5′-GCCGGCCCCTTTCCGCGTTA-3′, 20mer). A total of 71 open reading frames (ORFs) are predicted as coding sequences, which are divided into two regions with opposite transcriptional directions. Unfortunately, most of the predicted genes are still of unknown function. Thus, a more detailed investigation is required to fully understand the nature of this novel phage, and this was a major goal of the present study.

### Identification and characterization of the phage PaP3 terminase subunit

**Fig. 1:**
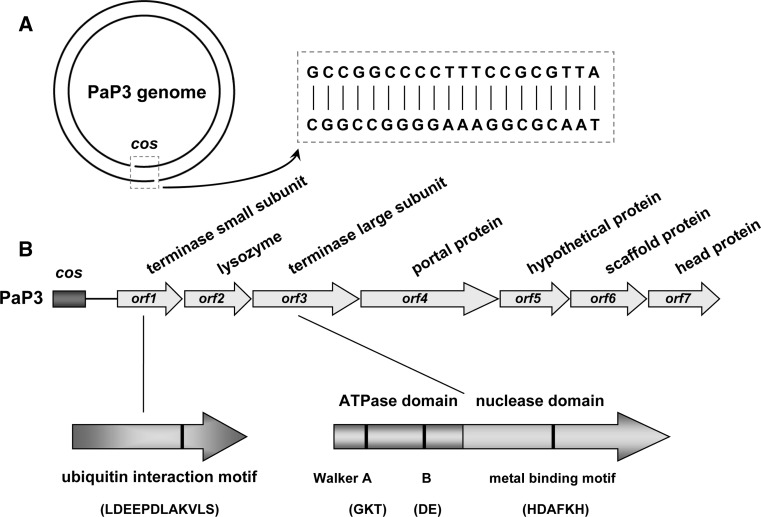
**Structure of** ***cos*** **and the genetic organization of the morphogenetic gene cluster of PaP3.** (**A**) The *cos* end of mature PaP3 genomic DNA. The 20-bp *cos* sequence is shown in the expansion. (**B**) Morphogenetic genes of the putative PaP3 late operon. Functional domains and predicted motifs of the PaP3 terminase subunits are indicated

**Fig. 2:**
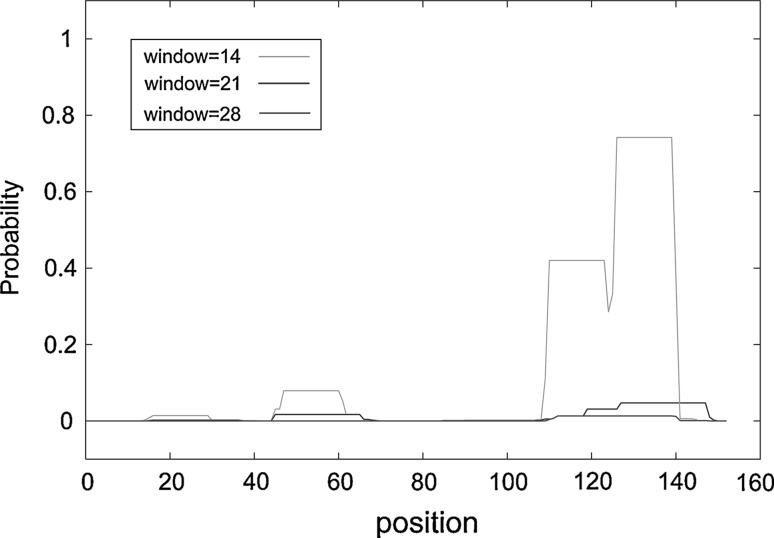
**Prediction of a coiled-coil motif (CCM) in p01 by COILS2.** Probability scores on a scale of 0 to 1 are plotted against the amino acid (aa) residue number. Peaks in the graph indicate regions of higher coiled-coil probability. The default output of probabilities in the scanning windows of 14, 21, and 28 aa residues are shown in green, blue and red, respectively. The region between residues 109 and 140 is predicted, with high probability, to form CCM

Based on sequence analysis, most of the morphogenetic genes of the putative PaP3 late operon are located on the left half of the phage genome (Fig. 1). The left-end gene *orf1*, which is located in the vicinity of the *cos* end of PaP3, codes for a 153-amino-acid (153-aa) protein with limited similarity (27.9 %) to the known small terminase subunit Xtma of a *Bacillus licheniformis* prophage [28]. No putative conserved domains have been detected using BLASTP-based tools. Most of the known small terminase subunits contain a predicted N-terminal helix-turn-helix (HTH) DNA-binding motif that is not only responsible for the recognition of viral DNA and subsequent initiation of packaging [27] but also involved in the specific binding of the *cos* site to prevent premature DNA release from the pressurized nucleocapsid upon completion of genome packaging [40]. However, extensive sequence analysis failed to predict such an HTH motif in the *orf1* gene product, suggesting that it may have a unique DNA recognition and binding pattern. Further sequence analysis using COILS2 (http://www.ch.embnet.org/software/COILS_form.html) revealed the likely presence of a coiled-coil motif (CCM) in the ORF1 protein (Fig. 2), which has been reported to be critical for protein dimerization or oligomerization [18]. This is consistent with the fact that a fraction of ORF1 exists as a dimer in solution (described below).

Adjacent to *orf1* is a putative lysozyme gene, which is hypothesized to be necessary for host cell lysis and release of mature phage [36, 42]. The gene immediately downstream of *orf2* encodes a 482-aa protein that exhibits 36 % identity to the large terminase subunit of *Enterobacteria* phage P22 [22], implying a similar function in DNA packaging. ORF3 is predicted to have an N-terminal ATPase domain that powers DNA translocation [27], which consists of a Walker A box (GKT) located at aa 54-56 and a Walker B box (DE) at aa 175-176. At its C-terminal region, ORF3 has a DNA cleavage domain predicted to generate the termini of packaged DNA [27]. In addition to the three leftmost genes, morphogenetic genes from *orf4* to *orf7* code for the portal protein, hypothetical protein, scaffold protein, and head protein, respectively. Among these, the portal protein is proposed to form the hole through which DNA is packaged into the prohead, and it is also a part of the packaging motor, while the scaffold protein assists in the assembly of the outer shell and dissociates from the capsid during subsequent DNA packaging [5]. To provide a preliminary insight into the PaP3 DNA packaging reaction, *orf1* and *orf3* were selected as candidate packaging-associated genes (based on sequence similarity to known packaging genes) and tested for terminase function.

### Expression and purification of phage PaP3 terminase proteins p01 and p03

**Fig. 3:**
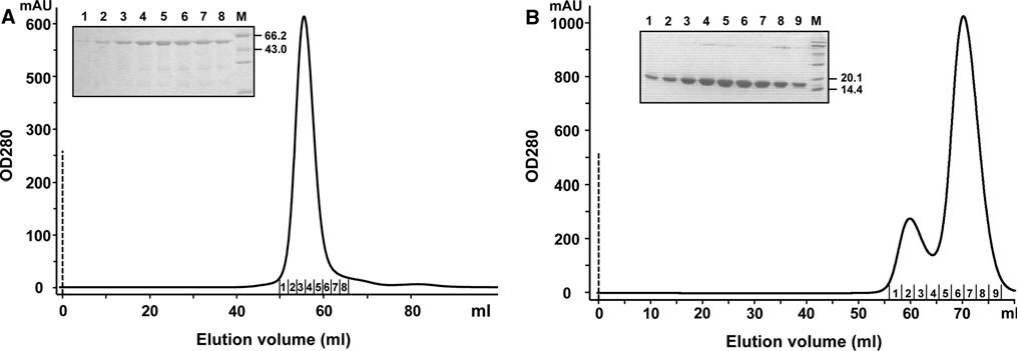
**Purification of the PaP3 terminase subunits by gel filtration.** (**A**) Elution pattern of the large subunit p03. The peak fractions (tubes 1-8) were electrophoresed on a 10 % polyacrylamide gel. Lane M shows a portion of the standard protein markers. (**B**) Elution pattern of the small subunit p01. The peak fractions (tubes 1-9) were electrophoresed on a 15 % polyacrylamide gel. Lane M shows the standard protein markers

Due to the potential interaction between the C-terminal region of the small terminase subunit and the N-terminus of the large subunit [27], the *orf1* and *orf3* genes were cloned and overexpressed in *Escherichia coli* as N-terminally and C-terminally His_6_-tagged proteins, respectively [14]. The protein products of interest were designated p01 and p03. Both p01 and p03 were efficiently expressed after induction at 25 ºC and found mainly in the soluble fractions of crude cell lysates. Highly purified proteins were prepared by Ni^+^ affinity chromatography followed by gel filtration on Superdex-75 columns. Figure 3 shows the elution profiles of these proteins from the size exclusion column. From the elution profile of p03, we estimated that its molecular weight (*M*_r_) is 56 kDa based on a number of protein standards (Fig. 3A), and this is consistent with the mass predicted from the sequence of the recombinant protein (*M*_r_ = 55.5 kDa). The single p03 peak in the chromatogram suggests that the recombinant PaP3 large terminase subunit exists in solution as a monomer, which is also the case for many other phages [13, 16]. In the case of p01, the protein eluted in two fractions: a minor peak corresponding to a higher-order oligomer, followed by a major peak as an apparent monomer (Fig. 3B). SDS-PAGE analysis of the column fractions revealed that each peak contains the p01 subunit. According to the elution position of the molecular-weight standards, the fraction centered at an elution volume of 60 ml is expected to correspond to a dimer. All purified proteins were estimated to be > 95 % homogeneous as judged by SDS-PAGE and were concentrated to 20 mg/ml using a Centricon device with a 10-kDa cutoff.

### The p03 large subunit exhibits a *cos*-dependent endonucleolytic activity

**Fig. 4:**
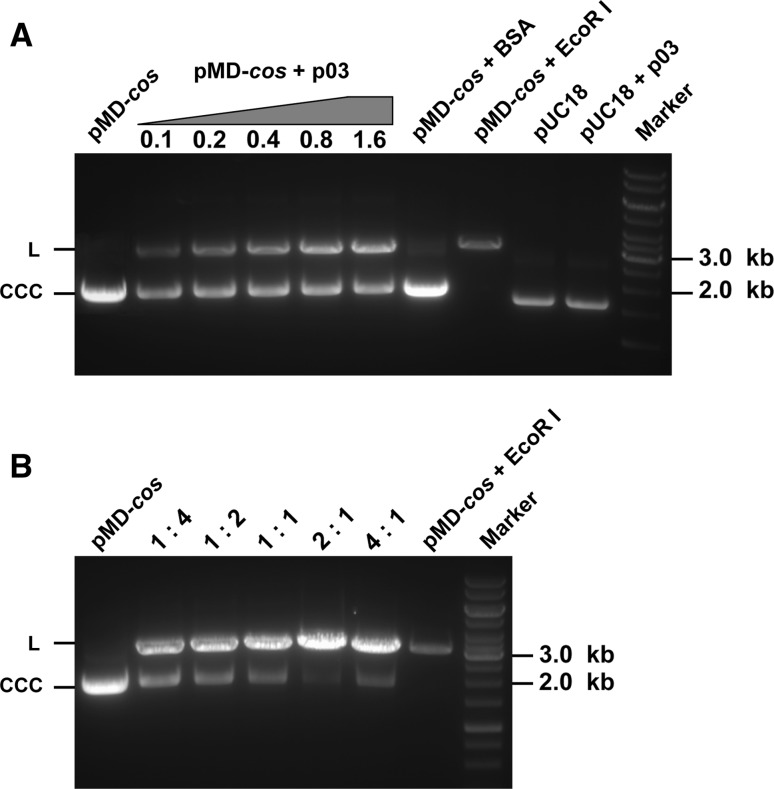
**Endonucleolytic activity of the large terminase subunit p03 and the stimulatory effect of the small subunit** **p01** **on this activity.** (**A**) Endonucleolytic activity of p03 in the absence of p01, using the *cos*-containing plasmid pMD-*cos* as the substrate. The concentrations of p03 are indicated (μM). The 1-kb DNA ladder marker is shown to the right (M). L, linearized plasmid DNA; CCC, covalently closed circular plasmid DNA. (**B**) Influence of different ratios of the small and large subunit (p01:p03) on endonucleolytic activity. The concentration of p03 was held constant at 0.8 μM

Because both initial and terminal cuts of the viral DNA substrate are required during phage packaging, the specific endonuclease activity of the large subunit of PaP3 terminase was tested using supercoiled plasmid (PMD-*cos*) containing the full *cos* sequence of PaP3. As shown in Fig. 4A, the endonuclease activity of p03 increased linearly with the concentration of terminase between 0.1 and 0.8 μM, with little increase at concentrations above 0.8 μM. The maximum efficiency of cleavage yielded approximately 45 % linear plasmid DNA compared to 3 % in the untreated control. The observation that not all DNA was linearized may imply that the endonucleolytic activity of the p03 large subunit needs the cooperation of the p01 small subunit. As expected, the endonucleolytic activity of p03 was significantly stimulated by addition of the p01 small subunit (Fig. 4B). We combined different ratios of the small and large subunit (p01:p03) and found that increasing proportions of p01 correlate with increasing endonucleolytic activity, with a visible decline at a ratio of 4:1. The molar ratio of p01:p03 for maximal effect was 2:1, yielding 92 % linearized product. No nuclease activity was observed for the small subunit p01 alone (data not shown). These results establish that the PaP3 large terminase subunit possesses a specific endonucleolytic activity on the PaP3 *cos* sequence, while the small subunit has a stimulatory effect on this activity. These two subunits may act cooperatively on the *cos* site of multimeric replicating PaP3 DNA and introduce staggered nicks to generate the 20-base ssDNA cohesive ends of the mature phage genome.

### Both the small and large subunit show specific DNA-binding activities

**Fig. 5:**
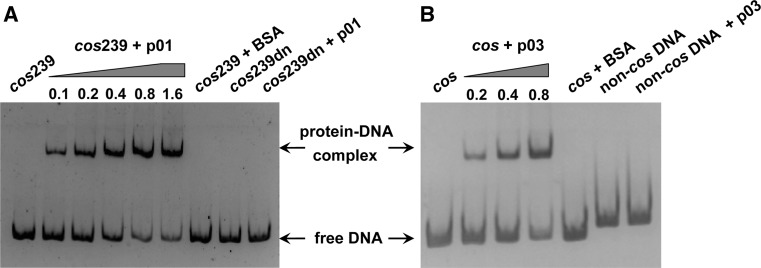
**Specific DNA-binding activity of the PaP3 terminase subunits.** (**A**) Binding of the p01 small subunit to DNA. A 239-bp DNA fragment containing the full PaP3 *cos* site (*cos*239) was incubated with increasing concentrations of p01 (0.1, 0.2, 0.4, 0.8, and 1.6 μM). The migration of the DNA without protein added is shown at left. A downstream 239-bp segment (*cos*239dn) was used as a nonspecific DNA control, and BSA was used as a nonspecific protein control. The samples were electrophoresed on a 5 % polyacrylamide gel in TBE buffer. (**B**) DNA-binding activity of the p03 large subunit. The 20-bp *cos* sequence was used as substrate DNA and incubated with 0.4 μM p03. A 24-bp non-*cos* DNA was used as a nonspecific control

In many phages, the small terminase subunits are involved in DNA binding *in vitro*, including λ gpNu1 with the *cos* site [23], SPP1 G1P and SF6 G1P with *pac* [7, 8], and T4 gp16 with the packaging initiation site [17]. These cases strongly suggest that PaP3 p01 may be also involved in *cos* site recognition and initiation of PaP3 packaging *in vivo*. Here, a 239-bp PCR fragment (*cos*239) containing the *cos* site of PaP3 was used as substrate DNA to investigate the DNA binding activity of the p01 small subunit. When the DNA target was mixed with p01 in binding buffer, a portion of the DNA displayed retarded mobility in a dose-dependent manner when compared to free DNA alone (Fig. 5A). In contrast, the p01 small subunit showed no binding activity when a nonspecific DNA of the same length (*cos*239dn) was used as a control, suggesting a sequence-specific binding activity of p01.

Next, we studied the DNA binding activity of the p03 large subunit to a 20-bp dsDNA target *cos* sequence. As shown in Fig. 5B, a clear band shift was observed for the *cos* DNA probe with increasing p03 concentration. As a control, no retarded mobility was observed for the nonspecific DNA in the presence of p03. Thus, these results demonstrated that both the PaP3 terminase small subunit p01 and the large subunit p03 have specific DNA-binding activity *in vitro*.

### Determination of the ATPase activity of the terminase subunits

**Fig. 6:**
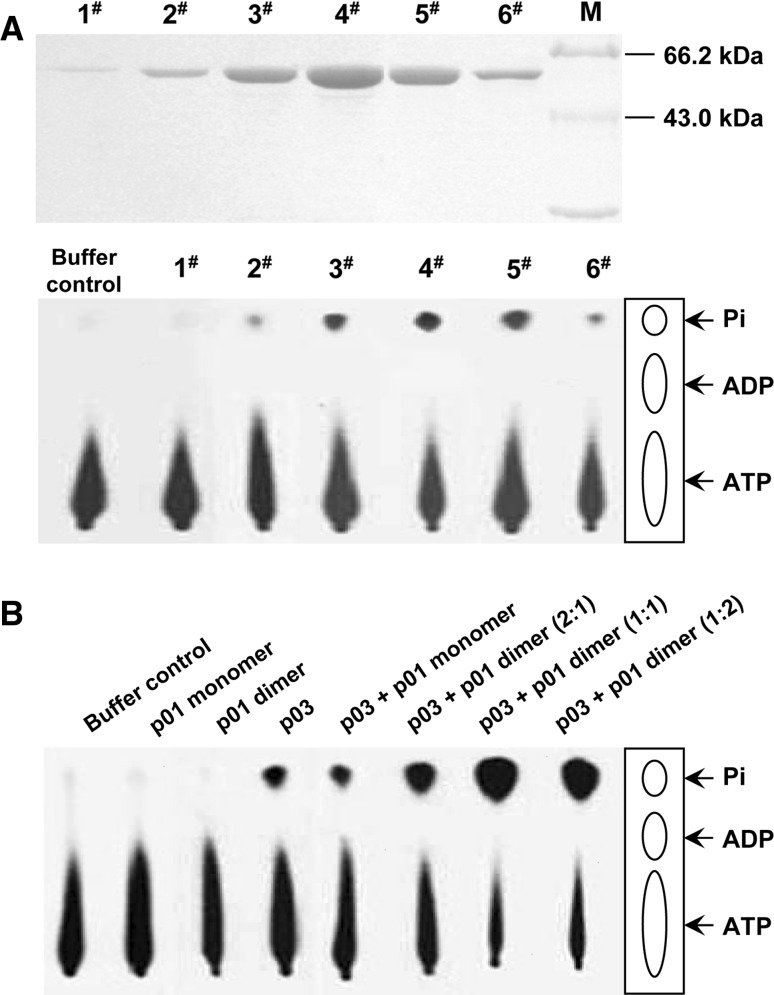
**ATPase activities of the PaP3 terminase subunits.** (**A**) The ATPase activity is inherently associated with the large subunit p03. The purified p03 was eluted with gel filtration buffer, and 1-ml fractions were collected. Each fraction was analyzed for protein by SDS-PAGE and for ATPase assays (the gel filtration buffer was used as a negative control). (**B**) The p01 dimer stimulates p03-ATPase activity. ATPase assays were performed using p03 alone (0.4 μM) or in the presence of monomeric or dimeric p01. The ATPase reaction buffer was used as a control

Previous studies have revealed that the ATPase activity that powers DNA translocation is generally located in the N-terminal domain of the large terminase subunit [27], and sequence analysis indicates that this is also likely for p03 (Fig. 1). To more fully explore this prediction, the ATPase activities of both the small and large subunit were analyzed. As shown in Fig. 6, the purified PaP3 large terminase subunit p03 exhibited the expected ATPase activity; incubation of p03 with [γ-^32^P]ATP resulted in the formation of inorganic ^32^Pi, suggesting that p03 hydrolyzed the β-γ phosphodiester bond, generating ADP and ^32^Pi. To determine whether the p03-ATPase activity is inherently associated with p03 as opposed to a minor contaminant ATPase in the preparation, the ATPase activities of p03 from the gel filtration column fractions were analyzed (Fig. 6A). The relative concentration of p03 in peak fractions 1 to 6 mirrored the ^32^Pi released in the ATPase assay, and the amount of ^32^Pi gradually accumulated with increasing concentrations of p03.

It has been documented that some small terminase subunits also contain a weak ATPase activity or stimulate the ATPase activity of the large subunits [3, 13, 39]. In numerous experiments, the PaP3 small subunit p01 alone did not show significant hydrolysis of ATP, regardless of whether the monomer or dimer was used (Fig. 6B). However, the p03-associated ATPase activity was enhanced in the presence of the p01 dimer, while the p01 monomer exhibited no significant stimulation of ATPase activity under the same conditions.

## Discussion

Phages have been a useful model system for studying assembly processes for over half a century. Much of our current knowledge about phage DNA packaging mechanisms comes from *Enterobacteria* phages (such as λ and T4) and *Bacillus* phages (such as φ29 and SPP1). DNA packaging in other phages has so far received limited attention. However, studying the packaging reactions of diverse phages both provides an opportunity to identify alternative mechanisms and can extend our understanding of the packaging process, as well as the formation and function of nucleoprotein complexes in general.

Although phage D3 is one of the relatively well-studied *P. aeruginosa* phages, its DNA-packaging mechanism still remains largely unknown [12, 30]. In the present work, we provide a relatively detailed functional characterization of the DNA-packaging terminase from *P. aeruginosa* phage PaP3. The DNA-packaging terminase of phage PaP3 is a multi-subunit complex composed of the small subunit p01 and the large subunit p03, products of the *orf1* and *orf3* genes, respectively. In nearly every tailed phage with a gene order that is known, terminase subunit genes are adjacent and located on the same DNA strand. However, some interesting exceptions exist. In the case of *Yersinia* phage PY100, ORF2 and ORF18, which encode the small and large terminase subunits, respectively, are widely separated and located on opposite strands [29]. Actually, during the initial annotation of the PaP3 genome, the leftmost gene, *orf1*, was predicted to encode a polypeptide of unknown function with similarities to a number of phage hypothetical proteins, not a small terminase subunit. This is due to the fact that the small terminase subunits of various phages display considerable sequence heterogeneity and distinct domain architectures. The previously identified terminase small subunits of phages T4 (gp16), P22 (gp3), SPP1 (G*1*P), SF6 (G1P), and λ (gpNu1) generally lack sequence similarity with one another [22].

Consistent with previous studies of other well-defined DNA packaging systems, the purified PaP3 terminase subunits possess typical properties such as ATPase, nuclease, and specific DNA-binding activities. However, two new observations were made during this study. First, the p03 large terminase was found to bind to DNA when it is unassembled. To our knowledge, this has not been observed previously. Second, a small terminase of a new type was informatically found and functionally identified. The lack of sequence similarity to other known small subunits and the absence of a typical HTH motif in the N-terminus suggest that it may have a unique DNA recognition and binding pattern in the PaP3 packaging reaction.

ATP hydrolysis is required *in vitro* for phage DNA packaging. ATP both provides an energy source for DNA translocation into the prohead and acts as an allosteric effector to control terminase holoenzyme specificity [15, 31]. Sequence analysis indicated that the PaP3 large terminase subunit has an N-terminal ATPase domain, which is consistent with our present experimental demonstration that p03 possesses an ATPase activity that is stimulated by the p01 small subunit. In T4, the large subunit displays a DNA-dependent ATPase activity [26], but the situation is different for PaP3 p03, which did not require the presence of DNA in our ATPase assays (Fig. S1). Terminase possesses ATPase catalytic sites that modulate the nuclease activity of the enzyme, drive its strand-separation activity, and power translocation during active DNA packaging [27]. In the *Bacillus* phage φ29, the packaging motor translocates 2 bp of DNA per ATP hydrolyzed and generates up to ~60 pN of force (a power density twice that of an automobile engine), similar to that measured with phage λ and T4, thus making the phage DNA packaging motor among the strongest biological machines reported to date [10, 11, 32].

In conclusion, we performed a preliminary functional characterization of the terminase from *P. aeruginosa* phage PaP3. The results presented here provide a necessary first step towards developing an *in vitro* DNA packaging system of PaP3. Detailed protein-protein interactions and biophysical studies are currently underway in our laboratory for a better mechanistic understanding of the complex assembly process of this interesting phage.

## Electronic supplementary material

Below is the link to the electronic supplementary material.

**Figure S1 The large subunit p03 displays a DNA-independent ATPase activity.** ATPase assays were performed using p03 (0.4 μM) with increasing concentrations of the PaP3 genomic DNA (Lanes 1-4 correspond to 0, 0.4, 4, and 40 nM of DNA, respectively). (TIFF 245 kb)
