# Supplementary figures and images for: Functional identification of the DNA packaging terminase from Pseudomonas aeruginosa phage PaP3
Source: Arch Virol. 2012 Jul 22;157(11):2133–41. doi: 10.1007/s00705-012-1409-5 (PMC3488191; doi:10.1007/s00705-012-1409-5)

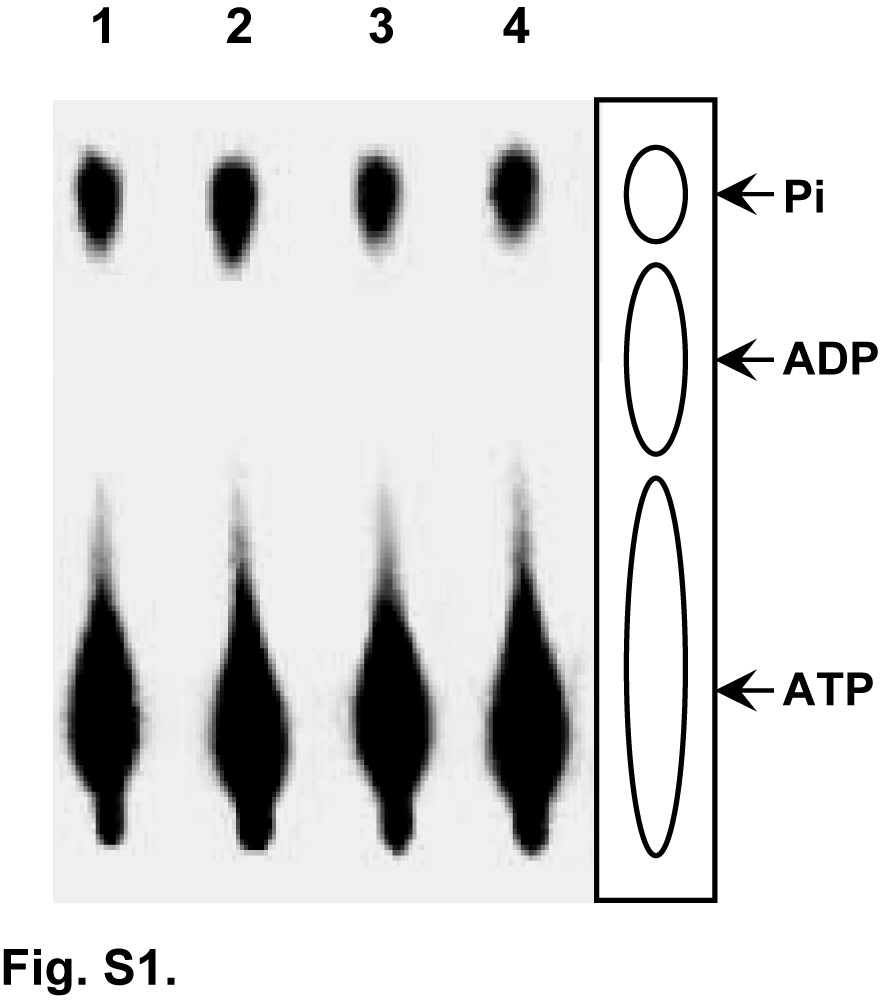

Supplement: Supplementary file 1 — Figure S1 The large subunit p03 displays a DNA-independent ATPase activity. ATPase assays were performed using p03 (0.4 μM) with increasing concentrations of the PaP3 genomic DNA (Lanes 1-4 correspond to 0, 0.4, 4, and 40 nM of DNA, respectively). (TIFF 245 kb) [file 705_2012_1409_MOESM1_ESM.tif]
